# Factors Influencing Informed Consent Preferences in Digital Health Research: Survey Study of Prospective Participants

**DOI:** 10.2196/63349

**Published:** 2025-01-23

**Authors:** Brian J McInnis, Ramona Pindus, Daniah Kareem, Daniela G Vital, Eric B Hekler, Camille Nebeker

**Affiliations:** 1 School of Information University of Texas at Austin Austin, TX United States; 2 Herbert Wertheim School of Public Health and Human Longevity Science University of California San Diego La Jolla, CA United States

**Keywords:** digital health, research ethics, informed consent, readability, health literacy, human-centered approach, consent communication, text snippet, qualitative analysis, effectiveness, health information, health informatics

## Abstract

**Background:**

Readability is important to consider when developing informed consent communications for prospective research participants, but not the most important consideration. Other factors to consider relate to learning preferences and literacy needs of people recruited to participate in research, as these factors can influence understanding of consent communications. To promote understanding among prospective participants, researchers should take a human-centered approach to develop consent communications.

**Objective:**

This study aims to explore how factors related to readability, topic, and participant demographic characteristics play into preferences for digital health research consent material. These factors are important to consider as not attending to some details that matter to a specific subgroup of prospective participants may systematically exclude people from research.

**Methods:**

People eligible to participate in a digital health study were recruited to review 31 paragraph length sections of a consent form, referred to as “text snippets,” for an existing institutional review board–approved digital health study. Participants (N=79) were surveyed and asked to choose between 2 variations of the text snippets, either indicating a preference for the institutional review board–approved original or a version that was modified to improve readability.

**Results:**

A slim majority of participants provided feedback about the snippets (n=44; 55%). Our qualitative analysis of the feedback found that participants preferred shorter snippets, in general, but the snippets also elicited new questions not addressed by the original consent material. This observation is supported by our quantitative analysis, which found that when the character length of the original was longer, participants were less likely to prefer the original (*P*<.001) and more likely to prefer the modified text by a factor of 1.20 times (*P*=.04), and particularly for snippets explaining study risks (*P*=.03). Our analysis also found significant differences in participant demographic characteristics. For example, older participants tended to prefer the original more than younger participants, by a factor of 1.95 times (*P*=.004). The results present illustrative examples of how factors related to sex, age, physical activity, and ethnicity all play into preference for consent communication.

**Conclusions:**

The findings point toward new ways of evaluating informed consent communication: (1) for responsiveness to specific prospective participant populations, and (2) effectiveness at eliciting informed questions from people considering participation. We discuss how creating partnerships with prospective participants to prototype informed consent materials, specifically study procedures and risks, can be a way to identify those details before launching a study. Furthermore, future research should go beyond “readability” to explore alternate measures of evaluating consent materials, such as the likelihood that the consent material and communication procedures will elicit “informed questions” for the research team.

## Introduction

### Background

The process of obtaining and providing informed consent to participate in research is a cornerstone of research ethics. The content of the consent form and its readability has been a topic of concern in health research and is well documented in academic literature [1-3]. Several peer-reviewed studies have focused on improving the ability of prospective research participants to comprehend these documents [4-6]. Specific to promoting the readability of consent materials, institutional review boards (IRBs) in the United States suggest the consent form be written at a sixth- to eighth-grade reading level. Aiming for the sixth- to eighth-grade reading level is a practical guideline with the goal of providing comprehensive information that is, ideally, accessible to most prospective participants. Researchers and IRBs try to follow the reading level guidelines and create consent communications that are clear, concise, and accessible to those who may consider participation. Consent forms that use plain language strategies, including shorter sentences and fewer syllables, appear to improve participant comprehension [7,8]. Consent forms that are not accessible compromise the goal of being informed and undermine the ethical principle of respect for persons [9,10].

Digital health research introduces new complexities to the process of communicating study information to prospective participants. Digital health research can involve data collection strategies and intervention delivery using tools like mobile apps, wearable sensors, social media platforms, and ecological momentary assessment. There is also growing attention on the use of health data repositories, including electronic health records, for training machine learning and other forms of artificial intelligence. These technologies support health research in many ways. For example, health data including sleep, heart rate, and step count can be collected passively in real time. Surveys assessing caloric intake, physical activity, and mental health can be deployed at scheduled or random times using an ecological momentary assessment app, reducing the need for recording data in a journal or relying on self-report [11] and personalized algorithms may be used to test strategies to motivate increased physical activities [12].

There are benefits associated with the use of digital health tools and strategies in health research, but there are also new risks associated with the volumes of granular health data produced and the potential inferences emerging from combining various data types. These risks involve data management protocols and privacy limitations that are important to convey to those considering research participation [10]. Moreover, many of the technologies used in digital health research are commercial products that may have data management processes and privacy protections that put a prospective participant at increased risk of harm. As with any health research, including clinical trials, some specialized knowledge may be needed to understand the study purpose and approach to data collection and management. It is also unlikely that people will have the time to obtain the foundational knowledge needed to facilitate truly informed consent. These tensions create additional challenges for digital health research. While a patient recruited to participate in a study may be somewhat knowledgeable about their specific health condition, they may not understand how the technologies used in digital health research gather, process, and store their data [10]. In fall 2023, the National Institutes of Health (NIH) posted a request for information specific to how informed consent may be improved for digital health research, which included sample language and points to consider [13]. This initiative by the NIH to assist the digital health research community in communicating complex digital health study information is consistent with the goals of our study reported in this paper.

This survey-based study was conducted to identify ways of improving consent communications in digital health research. Specifically, the study investigated the following research question exploring the relationship between the content of consent communications and prospective participant background characteristics on preferences for more and less information in consent material.

### Research Question

How do factors related to the content of consent material (eg, readability, length, and lexical diversity) and background characteristics associated with prospective participants (eg, sex, age, physical activity, and tracking physical activity) play into preferences for more and less information in an informed consent process?

This research received support from an NIH bioethics supplement to a parent study called “Your Move.” The “Your Move” study sought to test if a personalized digital health intervention that included several features including a smartwatch, a personalized adaptation algorithm called a controller, and a web-based self-experimentation tool, could help individuals improve their physical activity relative to providing digital health support that is not personalized and represents current deployed wellness program offerings. One challenge, when designing this study, was how to convey to prospective participants that a personalized algorithm would be used to nudge behavior change toward increased physical activity. This paper presents recommendations about how researchers can address these types of concerns, by making practical improvements to informed consent materials and procedures.

## Methods

### Participant Recruitment

Our study recruited participants who met the eligibility criteria to participate in the “Your Move” digital health study. Potential participants were recruited through a variety of sources, such as digital research portals (eg, ResearchMatch and Craigslist), via word of mouth through community partnerships with local university and public-school employee networks, as well as through digital advertisements.

Participant eligibility was the same as for the parent digital health study with the following inclusion and exclusion criteria.

#### Inclusion Criteria

Inclusion criteria were as follows: ability to read English; age of 25 years or older at enrollment; being physically inactive, which in the context of the study means less than 150 minutes (about 2.5 hours) of exercise per week; and the ability to access the internet through a smartphone, computer, or internet-connected device.

#### Exclusion Criteria

Exclusion criteria were as follows: participants develop a physical or mental health issue that prohibits compliance with the study’s protocol, participants become pregnant, and participants do not follow the instructions given to them by the research team.

### Ethical Considerations

This study was registered with the University of California San Diego IRB (#201720) and determined to be exempt as defined by the Common Rule (45 CFR 46.104(d)(2)). The consent process involved emailing prospective participants a summary of the study’s purpose and procedures, and a link to an interest survey. The digital interest survey included the full text of the consent communication describing study involvement on the first page, with a response item to indicate willingness to participate. Those who did not agree to participate automatically exited the survey. All participants were recruited during June 2022. The study team exported the collected data on a regular basis and stored it in a secure folder with limited access under the University of California San Diego Health Microsoft OneDrive environment. Storage conforms to the Health Insurance Portability and Accountability Act requirements. Completed surveys were manually deidentified by removing names and email addresses. After deidentifying the survey data, the list of participants was randomized by assigning everyone a random number in Excel. This list was shared via University of California San Diego Health Outlook email, utilizing encryption technology. Participants received a US $20 value Amazon.com gift card via email after confirming completion of the survey.

### Survey-Based Study Procedures

The survey-based study included three parts to elicit: (1) perspectives on information provided using standard consent processes, in general, (2) ratings to evaluate the clarity and comprehensiveness of specific snippets of consent information, and (3) demographic and background characteristics to consider the participant’s eligibility to participate and interest in the digital health technology study (eg, level of physical activity). Part 2 of the study involved reviewing 16 pairs of consent communication “snippets,” which were small sections of the consent form text. The snippets differed in terms of their assessed readability, specifically, each pair included a snippet of text from the IRB-approved consent form (hereafter the “original”) as well as a version of the original that had been improved for readability (hereafter the “modified”).

To create the modified variation of the original consent materials, three members of the research team independently used a free web-based readability analysis software, Readability Calculator, to rewrite the original text, while using the software to monitor how their edits played into specific measures, including the character length, Flesch Kincaid Reading Ease, and lexical density. All researchers then compared their modified text to each original snippet, and associated readability measures, to agree on a final version of the modified text that was the “most readable” based on the readability software (Multimedia Appendix 1). An illustration of a snippet before and after modification is evident in snippet 7 (Textbox 1), which describes the procedure for assessing participant heart rate with moderate to difficult walking conditions.

**Snippet 7 example of the original and modified versions of the consent material presented to participants.**

**Original text from the study consent form**

- “You will complete a treadmill walking test, while wearing a heart monitor on your chest, so that the research team can record your heart rate and blood pressure. The treadmill test will last approximately 5-10 minutes. The treadmill will start at a slow pace and gradually increase in speed and steepness, as though you are gradually walking faster up a hill. If you want to stop the test for any reason—such as if you feel lightheaded, dizzy, or breathless—a member of the research team will be there with you to help.”
- Flesch Kincaid Grading level of 10.52.

**Modified version of the consent material**

- “You will be asked to walk on a treadmill for 5-10 minutes, or as long as you can, while wearing a monitor on your chest to record your heart rate and blood pressure. A member of the research team will be there to help.”
- Flesch Kincaid Grading level of 8.92.

To confirm that the modified versions were, in fact, more readable than the original text based on standard measures, Table 1 presents descriptive statistics comparing the original and modified snippets, which indicates significant differences in terms of reading ease and character length. However, lexical diversity was not significantly different, which may be due to challenges in finding synonyms for technical and scientific terminology.

**Table 1:** Descriptive statistics related to text analysis variables.

			Mean (SD)		Maximum		ANOVA comparison between original and modified, *F* test (*df*)
**Original**							
	Flesch Reading Ease	49.70 (9.31)		64.59		—^a^	
	Character length	310.53 (151.81)		766.00		—	
	Lexical diversity	47.65 (5.77)		57.81		—	
**Modified**							
	Flesch Reading Ease	55.69 (13.81)		77.41		4.13 (1, 62)^b^	
	Character length	173.37 (91.57)		480.00		19.51 (1, 62)^c^	
	Lexical diversity	49.62 (10.21)		80.00		0.89 (1, 62)	

^a^No comparison.

^b^*P*<.01.

^c^*P*<.001.

During part 2 of the survey protocol, participants were instructed to compare the original and modified versions of 16 of 31 total snippets, which included 1 comparison that was evaluated by all participants (ie, “Item #01 Purpose: What is this study about?”). Specifically, participants were presented with the original text and asked the following questions.

- How relevant is the content to you (not at all, somewhat, very)?
- How clear is the content to you (not at all, somewhat, very)?

Then participants were presented with the original and modified texts, in a randomized order. Randomization was used to account for any potential ordering effect, which may influence the degree to which participants engage with the materials. For each snippet pair, participants were instructed the following.

“The following are 2 variations on the consent language. Please indicate the variation that you prefer,” using the options: (1) prefer version 1; (2) prefer version 2; (3) both versions 1 and 2 look good; and (4) both need improvement.

While options 1 and 2 directly contrast the modified and original options, options 3 and 4 allow for the possibility that the participant did not perceive enough of a difference between the 2 options. For consistency, throughout the paper we refer to preferences for either the original, modified, or both versions (option 3) as “good,” and both versions needing improvement (option 4) as “need.” From a methodological perspective, including options 3 and 4 also reduces the likelihood of guessing, which otherwise would add noise to the analysis. After comparing the original and modified snippets, participants were asked “What changes would you recommend to the text above?” to capture additional information about their perceptions of the content.

The snippets were labeled as either about the study purpose, procedures, risks, or benefits. All participants (N=79) reviewed a single snippet about the digital health technology study purpose. While all participants reviewed a total of 15 additional snippets, when they initiated the survey, they were randomly assigned to either read a set of snippets that included more risk items (called “survey A”) or more procedural items (called “survey B”). The random assignment resulted in 41 (52%) participants receiving survey A and 38 (48%) participants receiving survey B. We found no significant difference in the distribution of participant preferences for the original and modified versions of the snippets (*χ*^2^_6_=5.9; *P*=.42; Table 1). This indicates that the responses can be evaluated together in a series of logistic regression analyses. During the logistic regression analysis process, we considered including a variable to capture the effects associated with the survey A and survey B groups, but we found that including this variable did not significantly improve model performance and explainability.

### Mixed Methods Data Analysis

To investigate factors that increase the likelihood of participants preferring the original or modified versions of the consent snippets, a series of logistic regressions were fit to evaluate each preference independently: that is, prefer original, prefer modified, both are good, and both need improvement. A logistic regression is used to evaluate the log odds of a condition being true or false, where model A presents the log odds of preferring the original (“true”) over all other options (“false”).

To investigate how factors related to the content (eg, readability, length, lexical diversity) and background characteristics associated with prospective participants (eg, sex, age, physical activity, and tracking physical activity) play into preferences for the original or modified versions, we applied the survey data and reading analysis tools to construct a series of independent measures for the logistic regression analysis. Each model was fit by beginning with a beyond optimal set of the independent predictors, and then measures of overall “goodness of fit,” including a chi-square test of the model with and without predictors, were used in conjunction with tests to evaluate the effect of each predictor on the model, such as the Wald test, to select predictors for each final model. This iterative process helped to identify potential confounding factors in the analysis. As an example, initially, our team considered a broader range of readability measures (eg, Gunning Fog Index, Flesch Kincaid Grade level, Automated Readability Index); however, we found that many of the measures were highly correlated. To address this, we selected readability measures that reflect a range of important considerations in writing and that are not well correlated (Table 2).

**Table 2:** Correlations between text analysis variables.

			Original							Modified				
			Flesch Reading Ease		Character length		Lexical diversity		Flesch Reading Ease			Character length		Lexical diversity
**Original**														
	Flesch Reading Ease	1.00		–0.21		–0.12		0.20			0.06		0.09	
	Character Length	–0.21		1.00		0.08		–0.12			0.54		–0.05	
	Lexical Diversity	–0.12		0.08		1.00		–0.20			0.08		0.69	
**Modified**														
	Flesch Reading Ease	0.20		–0.12		–0.20		1.00			–0.31		–0.14	
	Character length	0.06		0.54		0.08		–0.31			1.00		–0.09	
	Lexical diversity	0.09		–0.05		0.69		–0.14			–0.09		1.00	

In the findings, the logistic regressions table presents the estimate and standard error for each predictor in each model. To improve the interpretation of the models, the inline description of the analysis in the Results presents odds ratios (ORs), which are the predictor estimates exponentiated. The raw estimates are on a logit scale, which is from negative infinity to positive infinity, but when exponentiated the predictor OR reflects the effect of the predictor on the probability of the dependent measure occurring. For example, in model A the effect of sex male on the preference for the original snippet is –0.68, which when exponentiated as OR is 0.50. As the OR of 0.50 is less than 1, the interpretation is that participants who identify as male are 0.50 times less likely than female participants to prefer the original snippet. As another example, the estimate for sex male in model C is 1.35, which when exponentiated translates to an OR of 3.84, implying that male participants are 3.84 times more likely to feel that both snippets are good enough than female participants. For measures of readability, the interpretation reflects a one standard deviation change in the predictor, for example, in model A an increase in the modified snippet character length by one standard deviation (estimate=–0.39) contributed to a decrease in the likelihood that participants preferred the original snippet by a factor of 0.67 times.

## Results

### Review of Participant Demographics

In total 79 of 118 (67%) initiating the survey completed the study. Just more than half of the participants identified as female (n=41, 52%), most were under the age of 40 years (n=51, 64%), and 16 participants identified as Hispanic or Latino (20%). The participants reported a range of self-assessed physical activity. The number of people rating themselves as “more active than average” (n=31, 39%) was roughly equivalent to those rating themselves “less active than average” (n=32, 40%). A third of participants also indicated that they track their physical activity, which is important to note as the study context involves a digital health technology intended to promote an active lifestyle (Table 3).

**Table 3:** Descriptive statistics related to participant backgrounds (N=79 participants).

			Value, n (%)
**Sex**			
	Female	41 (52)	
	Male	38 (48)	
**Age (years)**			
	Under 30	23 (29)	
	30-39 years	28 (35)	
	40-49 years	17 (21)	
	50-59 years	7 (9)	
	60 years or older	4 (5)	
**Ethnicity (Hispanic)**			
	No	63 (80)	
	Yes	16 (20)	
**Physical activity**			
	Average	16 (20)	
	Less active	32 (40)	
	More active	31 (39)	
**Track physical activity**			
	Yes	25 (31)	
	No	54 (69)	

The majority preferred the original to the modified version improved for readability, then both are good enough. In just 6 comparisons, participants preferred the modified version, which was improved for readability, to the original IRB-approved text (Table 4). To investigate why participants may have preferred the modified version to the original and vice versa, we first reviewed feedback from participants. Of the 79 participants, 44 (55%) provided feedback related to the snippets, and those who provided feedback provided a fair amount (mean 6.5, SD 5.24 responses per participant).

**Table 4:** Preferences by snippet and consent form topic^a^.

Preferences for the consent form snippets	Original, %	Modified. %	Good, %	Need, %
Benefits	36.90	26.19	32.74	4.17
Procedures	35.29	23.11	33.61	7.98
Purpose	34.15	27.44	29.88	8.54
Risks	33.72	26.05	31.16	9.07

^a^Percentages are based on the total number of snippets reviewed per topic. Specifically, the “benefits” section included 4 snippets, “procedures” included 12, “purpose” included 3, and “risks” included 11. Overall, participants tended to prefer the original version of the snippets; however, our qualitative analysis of the feedback and quantitative analysis of preferences by subgroup indicate that there are many additional factors to consider when developing a consent communication.

In snippet comparisons where participants preferred the modified version, participants shared 55 responses (31 unique participants). Most responses indicate that they preferred the modified version because it was shorter and less confusing than the original version, “[original] has too much text about eligibility. I prefer [modified] because it was more concise: If you are eligible...” [Participant 57] and “[original] too many numbers thrown at me-way way too wordy” [Participant 16]. However, participants also requested additional details that were not included in either version. Specifically, the 55 responses included 19 follow-up questions for the researchers, such as “Is it a licensed health care professional conducting the exam?” [Participant 28], “Maybe clarify how points are earned using an example” [Participant 26], and “[...] do I have to take time off work?” [Participant 25]. While participants appreciate brevity, the consent snippets elicited a range of follow-up questions for the research team.

For the most part, participants preferred the original version (in 14 comparisons) and shared a total of 140 responses (made by 40 unique participants) about their preferences. In these cases, many participants felt that the modified text was too short and “[...] leaves too many questions” [Participant 25]; though participants also made specific suggestions about how to trim the original version, “I think everything after measuring your blood sugar level is not necessary” [Participant 06] and “too much jargon” [Participant 47]. In some instances, the modified text was missing key details, “[modified] does not state that the treadmill will start at a slow pace and gradually increase speed. That is an important detail missing from [the] text” [Participant 23]. In general, participants preferred a trimmed-down version of the original, rather than the modified version.

The desire for more information was especially strong in response to snippets about study procedures and risks. While participants tended to prefer the original version to the modified version, they also identified many missing details, such as, whether they would be disqualified if stopping during a physical fitness examination involving a treadmill. However, participants did not raise any questions about the time commitment, retracting consent, removal from the study, costs associated with participation, and contact information for the research team. For a research team, knowing when to add and remove details from a consent document can be challenging, if they have yet to share drafts with prospective participants.

In 11 of the comparisons, participants indicated that they had no preference between the original and modified, that is, both are good. Participants shared 94 responses about these snippet comparisons (31 unique participants). In these cases, participants felt that both the original and modified versions had value that could be combined, “[modified] is more clear, but does not include the daily survey requirements listed in [original]” [Participant 40] and “[modified] needs to be more specific on what it means to have mental health issues, [but the original] has a clear understanding” [Participant 43].

Several participants suggested ways to rewrite the snippets to promote specific values in the communication, “combine them to remain positive: Our staff will show you how to wear the Fitbit so that it is comfortable. Our staff will show you how to wear it, help you adjust if needed, and provide an (optional) tutorial” [Participant 17]. However, 1 participant was just not sure what to suggest, “This just sounds awkward to me. I don't know how to improve. Maybe even deleting it completely” [Participant 6]. While each version of the consent material could be improved, in most comparisons, participants preferred the original version or felt that the modified and original could be combined.

### Risks and Readability, but Inconclusive

To explore how factors related to the consent communication content play into participant preferences for the original or modified versions, we conducted a logistic regression analysis of each preference category: that is, prefer original, prefer modified, prefer both, prefer neither. Presented in Table 5, our analysis evaluated how various factors related to content (eg, total character count including spaces and punctuation, reading level), as well as participant background characteristics (eg, sex and physical activity), may play into consent communication preferences.

**Table 5:** Logistic regressions to evaluate how aspects of the study and participant backgrounds relate to preferences. Note that all 79 participants responded to snippet 1, then 41 participants responded to 14 items, and 38 participants responded to 15 other items (a total of 15 items for all participants)=1223 responses.

			Model A: prefer original^a^							Model B: prefer modified^b^							Model C: both are good^c^							Model D: neither^d^				
			Estimate		SE		*P* value		Estimate			SE		*P* value		Estimate			SE		*P* value		Estimate			SE		*P* value
Intercept			0.02		0.85		—^e^		–0.87			0.90		—		0.43			0.93		—		–9.54			1.68		<.001
**Consent section (by comparison to “benefits”)**																												
	Procedures	–0.06		0.22		—		0.19			0.24		—		–0.23			0.25		—		0.45			0.47		—	
	Purpose	–0.11		0.28		—		0.18			0.29		—		–0.05			0.31		—		0.28			0.57		—	
	Risks	–0.28		0.24		—		0.59			0.26		.01		–0.55			0.26		.01		0.89			0.49		<.05	
**Sex**																												
	Male	–0.68		0.14		<.001		–0.47			0.16		.001		1.35			0.16		<.001		–0.20			0.26		—	
**Age (years)**																												
	30-39	0.35		0.19		.05		–0.19			0.20		—		–0.38			0.22		.05		0.39			0.40		—	
	40-49	0.67		0.23		.001		–0.15			0.25		—		–1.49			0.37		<.001		0.72			0.47		—	
	50-59	0.29		0.31		—		–1.07			0.35		.001		–2.98			1.03		.001		3.44			0.46		<.001	
	60 or older	–0.30		0.18		.05		–0.95			0.20		<.001		0.77			0.17		<.001		1.08			0.33		<.001	
**Ethnicity (Hispanic)**																												
	Yes	–0.23		0.16		—		0.22			0.17		—		–0.30			0.19		—		0.88			0.26		<.001	
**Physical activity**																												
	Less active	–0.13		0.18		—		–0.77			0.20		<.001		0.63			0.24		.001		1.70			0.41		<.001	
	More active	–0.72		0.19		<.001		–0.26			0.20		—		0.92			0.22		<.001		0.79			0.38		<.01	
**Track physical activity**																												
	Yes	–0.03		0.15		—		–0.91			0.17		<.001		0.66			0.18		<.001		0.96			0.28		<.001	
**Original**																												
	Character count (scaled)	0.17		0.08		.01		0.15			0.08		.05		–0.32			0.10		.001		–0.13			0.15		—	
	Lexical diversity	0.01		0.02		—		–0.02			0.02		—		–0.02			0.02		—		0.05			0.03		—	
	Reading ease	0.01		0.01		—		0.00			0.01		—		–0.02			0.01		.05		0.00			0.01		—	
**Modified**																												
	Character count (scaled)	–0.39		0.09		<.001		0.19			0.09		.01		0.19			0.10		.05		0.20			0.15		—	
	Lexical diversity	–0.02		0.01		.05		0.02			0.01		.05		0.00			0.01		—		0.01			0.02		—	
	Reading ease	–0.01		0.01		—		0.01			0.01		.05		–0.01			0.01		—		0.02			0.01		<.01	

^a^Goodness-of-fit *χ*^2^_18_=142.0; *P*<.001.

^b^Goodness-of-fit *χ*^2^_18_=88.9; *P*<.001.

^c^Goodness-of-fit *χ*^2^_18_=340.9; *P*<.001.

^d^Goodness-of-fit *χ*^2^_18_=101.2; *P*<.001.

^e^No significant effect.

A primary observation is that character count (scaled) significantly affects a participant’s preference for the original version of the consent material. Specifically, for every SD increase in the character length for the modified version, there is a corresponding decrease in the likelihood that participants prefer the original version, by a factor of 0.67 times (*P*<.001), and a corresponding increase in the likelihood that they prefer the modified version, by a factor of 1.20 times (*P*=.04). This directly responds to participant feedback that they tended to prefer the readability of the modified versions, though appreciate when additional details are provided. Other readability factors, including lexical diversity and Reading Ease, did not significantly affect participant preferences in our analysis.

Our analysis also found that the consent section (eg, purpose, procedures, risks, and benefits) played into participant preferences. Specifically, participants were slightly more likely to choose the modified version when presented with information about study risks, by a factor of 1.8 times (*P*=.03), and less likely to select that “both versions 1 and 2 look good,” by a factor of 0.57 times (*P*=.04). An initial takeaway is that participants tended to prefer risk communications that are more readable. As the results are marginally significant, future research should investigate the specific study risk topics that prospective participants would like described in more and less detail, rather than simply comparing differences between risks and benefits, as is the case in our analysis.

### Sex, Age, Physical Activity, and Tracking Physical Activity

Readability in consent communication, particularly character count, is an important consideration; however, our analysis highlights other factors related to the participants, specifically their demographic and background characteristics, that play into their consent communication preferences. Participants who identified as male were significantly more likely to indicate that “both versions 1 and 2 look good,” than participants identifying as female, by a factor of 3.84 (*P*<.001). This observation is useful to note, as female participants may have had stronger opinions about consent communication. Specifically, female participants were significantly more likely to choose between the original and modified versions, by a factor of 1.96 times (*P*<.001) and 1.60 times (*P*=.003). Future studies investigating consent communication content should carefully consider how sex identity may factor into preferences.

Additionally, our analysis found differences by age group. While participants in our study, grouped between the ages of 20-29 and 30-39 years were not statistically different in their preferences for consent communication, participants aged 40 years and older wanted different considerations. Specifically, participants aged 40-49 years tended to prefer the original version more than younger participants, by a factor of 1.95 times (*P*=.004) and were significantly less likely to state that both versions look good, by a factor of 0.22 times (*P*<.001). While our study recruited relatively few participants in the age ranges of 50-59 years (n=7, 8%) and 60 years and older (n=4, 5%), they tended to not prefer the modified, rather feeling that both versions needed improvement, in comparison to younger participants. As the specific digital health study used as a context for our research involved physical exercise and cardiovascular risks, prospective participants who are older in age may have preferred the original version of the consent materials because it included more detail. Future research should explore this trend further by recruiting older adult participants.

Study participants were asked about their level of physical activity, as people with already high or already low levels of activity may value different levels of detail about the study. By comparison to people who rate their physical activity as average, our analysis found that participants who perceive their activity as lower-than-average were less likely to prefer the modified version, by a factor of 0.46 times (*P*<.001), whereas participants with higher-than-average physical activity were less likely to prefer the original versions, by a factor of 0.48 times (*P*<.001). Our interpretation is that these differences in what participants do not prefer points toward general feelings that both versions were missing details that are relevant to the 60% of study participants (n=48) who rated their physical activity as higher or lower than average; however, these groups may need different levels of study detail.

Participants were also asked about their physical activity and tracking behaviors, as the digital health study context would involve wearing a smartwatch fitness tracking device. Our analysis found that participants who already track their own physical activity were less likely to prefer the modified version than participants who do not track their physical activity, by a factor of 0.40 times (*P*<.001). By comparison, participants who do not track their physical activity preferred the modified version, by a factor of 2.49 times (*P*<.001). While recommendations about how to communicate study details tend toward providing as much detail as possible, in our study the participants tended to prefer a pared-down version.

Finally, participants identifying as Hispanic or Latino felt that both versions of the consent communication need improvement, by a factor of 2.40 (*P*<.001). This is likely due to an oversight in our study design, as both versions of the content were presented in English, and participants were not offered a translated version. Despite this oversight in the study design, the strong signal in our analysis should be taken as a reminder to pay attention to the language needs of participants. In general, preferences related to the option “both need improvement,” deserve further consideration as our analysis found a significant effect in the intercept term for model D, which points to additional unexplained variation in the model.

## Discussion

### Principal Results

Researchers have an ethical obligation to provide information about the technologies used in digital health research, including related risks, benefits, and risk mitigation strategies. This information exchange within the context of health research currently occurs via the informed consent process. Standard recommendations for designing a consent communication encourage researchers to provide considerable details about all aspects of a study, which can yield a consent communication that is lengthy and dense.

Our study investigated the research question: How do factors related to the content of consent material (eg, readability, length, and lexical diversity) and background characteristics associated with prospective participants (eg, sex, age, physical activity, tracking physical activity) play into preferences for more and less information in an informed consent process? While in the aggregate participants in our study preferred the original IRB-approved consent language, our qualitative and quantitative analyses demonstrate that participants often missed information that seemed to matter to specific prospective participant subgroups for the study. Our analysis also found that the snippets elicited many informed questions about the hypothetical research, underscoring the importance of partnering with prospective participants to plan and finalize study materials.

Partnering with prospective participants to identify how to improve the research informed consent process has led to understanding the study information they most need and want. For instance, Koh et al [5] found that research participants were underinformed and prioritized information about risks or discomforts and study procedures. Similarly, McCarty et al [14] found that the 2 concepts research participants struggled with understanding were the experimental aspects of the study and who was responsible for paying for research-related injuries.

In general, our analysis found that participants prefer brevity but need details that correspond with their background and experiences related to the study. For example, participants who were older preferred the more detailed original version of the consent, whereas participants who identified as being younger than 40 years of age preferred the modified version. These differences may relate to the perception of physical risks associated with study procedures. However, lower-risk study procedures, such as tracking your physical activity with a smartwatch, may not need as much detail, as participants who do not currently track their activity tended to prefer the modified version of the consent. These and other findings point toward different information to offer for each subgroup in the study population, by sex, race, and ethnicity, as well as physical health, and technology adoption.

Prospective participants could help to identify these gaps. A slim majority of participants shared feedback about the consent material (n=44, 55%). While participants by and large preferred the original, many saw value in the way that the content was communicated through the modified consent material and suggested that the two could be combined. Partnering with prospective participants in research could create opportunities for researchers to identify these gaps early in the study design process, by sharing ideas and asking prospective participants for feedback [10]. As participants in our study reflected on ways to improve the snippets, they shared feedback that included questions related to study procedures, expectations, and risks.

The informed consent process is a starting point for people to cultivate a trusting relationship with researchers. Regardless of whether they choose to participate in a study, trust is likely cultivated when people feel as though they can have the opportunity to fully grasp study materials and raise questions with the research team. As algorithmic decision-making plays an increasingly central role in digital health studies, cultivating trust among all the people involved in research becomes ever more important as, without trust and shared understanding, research quality will be compromised. Researchers should consider actively partnering with study participants so that together they can work through any interesting outcomes and misalignments that may emerge through the algorithmic decision-making in a study.

### Recommended Practices for Working With Prospective Partners to Develop Trustworthy Consenting Processes

Many of the questions raised by participants focused on details not included in the original consent materials. By listening to prospective participants, research teams could identify barriers to participation that could be mitigated prior to recruitment [10]. While speculative, we contend that this could pay off in the long run-in terms of improved recruitment and retention based on increased confidence in the research team and understanding of what participants are committing to. In this way, the text snippets taken from the consent form offered a foundation for prospective participants to raise informed questions about the study, thereby enabling them to participate as guides of the research.

Researchers can take a few simple steps to incorporate participants as partners in guiding the study and the consenting process. Researchers can present prospective participants with a sample of the study consent materials to identify the unique needs and considerations of specific subgroups within the sample population. Our findings demonstrate that demographic background, susceptibility to possible risks, and study interest all play into a participant’s need for more and less detail about a study. Similar findings have been reported in previous studies, which found that ethnicity, level of education, age, and gender were factors impacting participants’ comprehension of informed consent information for genetics research, while factors like substance dependency were significantly more likely to increase the level of consent participants accepted [5,15,16].

By taking a 2-step approach to consent material development, digital health researchers can potentially identify these considerations and incorporate them to refine the study materials.

- Step 1: Broadly sample prospective participants for their perspective on snippets of the draft consent materials. At this stage, a sample of the prospective participants is presented with specific sections of the consent materials to comment on to elicit feedback on the language and any questions about the study details. Like our study design, the results should help to shed light on any differences based on demographic, interest-based, and other participant characteristics. Differences may include preferences for level of detail and methods of communication.
- Step 2: Adapt the consent materials for prospective participants. By surveying a sample of prospective participants about their preferences and questions about the consent materials, researchers will learn about any gaps and questions that need to be resolved in general, and for specific participant subgroups (eg, older adults, early technology adopters). This information can be adapted and integrated into broader consent processes for a study.

Our analysis shows how prospective participants in a study are not a monolith but reflect a diverse collection of subgroups that require specific considerations when supporting their learning about a study. Specifically, sex, age, level of physical activity, and ethnicity all played into preferences for more detail in the consent material. Researchers and IRBs should strive to identify and promote these considerations with research sponsors, to encourage the research funding necessary so that all participants may feel informed through research consent processes. The findings point toward new ways of supporting prospective participants through their process of making an informed decision about whether to participate in a research study. While these recommendations encourage more work on the part of researchers, we contend that the benefits have the real potential to outweigh the added time in the form of improving trustworthiness between researchers and participants. This has the likelihood of producing several benefits overall including improved recruitment, retention, and, likely most critically, the quality of science being conducted as prospective partners can help researchers to understand implicit assumptions of risks, questions, and opportunities that they may not have otherwise been aware of.

### Limitations

There are several limitations associated with this study. First, the research involved just 1 digital health study context, which was a low-risk smartwatch intervention to boost physical activity over a 1-year period. Future research should vary the study context to explore how higher risk and more complex study procedures, data management protocols, and digital health systems might play into prospective participant preferences for consent material. In particular, the use of artificial intelligence in health research is still novel and the implications are not fully understood. Researchers should explore best practices for communicating the details of a study involving such novel technologies.

Second, the study involved a survey-based approach to investigate participant perspectives on 31 text-based comparisons related to different aspects of the consent material. In some cases, the points of comparison were more similar than different, in other cases the opposite was true. In some cases, participants clearly agreed that more details were necessary on specific topics. Future research should explore various ways of communicating in more detail on specific topics, such as study risk. Rather than applying a survey-based approach, future research should aim to understand how people process this information by applying approaches like a cognitive interview, in which participants are asked to talk through their thought processes as they review the material. Rather than present 2 points of comparison, researchers should evaluate a wider range of options that include text and non-text-based methods of communicating consent materials [10]. Finally, readability is an important consideration when developing materials, but as demonstrated in this study the measures of readability selected for the analysis did not meaningfully impact prospective participant preferences related to the consent material. There may be other measures of the consent content that would provide meaningful ways of identifying content that is readable, useful, accessible, considerate, and so on. Our own analysis highlights how consent content can elicit informed questions, which may be a future measure of consent communication effectiveness.

### Conclusions

Readability has been a primary metric for evaluating the effectiveness of consent communication materials; yet, our study demonstrates that there are other considerations for researchers to weigh. Our analysis highlights that people prefer short and simple text descriptions of study details, but also want answers to questions that relate to their personal circumstances. This finding is based on the breadth of personal questions elicited through the study as well as quantitative analyses demonstrating that factors related to participant sex, age, level of physical activity, and ethnicity all played into preferences for more detail in the consent material. The paper presents a 2-step approach that researchers can take to investigate how such factors may play into consent communication design for a digital health research study. Future research should explore ways to incorporate people and communities into the development of consent communication, to promote understanding and partnership in science.

## Appendix

Multimedia Appendix 1 Snippets of the original and modified versions of the consent material presented to participants.

## Abbreviations

IRB: institutional review board
NIH: National Institutes of Health
OR: odds ratio
