# Supplementary material for: Factors Influencing Informed Consent Preferences in Digital Health Research: Survey Study of Prospective Participants
Source: J Med Internet Res. 2025 Jan 23;27:e63349. doi: 10.2196/63349 (PMC11803319; doi:10.2196/63349)
Supplement: Multimedia Appendix 1 [file jmir_v27i1e63349_app1.pdf]

Supplemental File – Original IRB approved consent text with modified consent text

Purpose

<table>
<tr><td colspan="3">Snippet #01: What is this study about?</td></tr>
<tr><td colspan="2">Original 40%</td><td>Modified 24%</td></tr>
<tr><td colspan="2">Dr. X from UXXX and some of his colleagues (the “research team”) are leading a research study to investigate whether a new type of digital health tool can encourage adults (25+ years old) to increase their average level of activity on a weekly basis over a 12-month period. The research team hopes that the study will produce new insights about how technology can be used to promote an active and healthy lifestyle. Participation in this study may or may not benefit you directly. If you are interested in participating in this research, please read this form carefully.</td><td>Dr. X and his team are leading a study to investigate if a new app for your smartphone and smartwatch can help adults increase their physical activity. The study will last 12 months.</td></tr>
<tr><td colspan="2">Preferences: Overall original was preferred</td><td>Feedback</td></tr>
<tr><td>Both 28%</td><td>Neither 9%</td><td>29</td></tr>
</table>

<table>
<tr><td colspan="3">Snippet #02: What is this study about?</td></tr>
<tr><td colspan="2">Original 31%</td><td>Modified 36%</td></tr>
<tr><td colspan="2">The following sections provide detailed information about each stage of the research, possible risks, benefits, and other considerations. Please feel free to ask questions before signing this consent. Our research team is available to answer any questions and address any concerns you may have at any point before, during, and after the study. Additionally, we encourage you to speak with other people, like family or friends, about your decision. At the end of the form, you will be asked if you consent to participate in the study. Your participation in this research is entirely voluntary, you can also say “yes” now, but change your mind later and if you say “no” we will not hold your decision against you.</td><td>Please read on for details about the study. Our team is happy to answer your questions and encourage you to talk with your family and friends about the study. If you decide that you want to participate, know that you can stop participating at any point.</td></tr>
<tr><td colspan="2">Preferences: Both versions emerged as good overall</td><td>Feedback</td></tr>
<tr><td>Both 26%</td><td>Neither 7%</td><td>9</td></tr>
</table>

<table>
<tr><td colspan="2">Snippet #03: What is this study about?</td></tr>
<tr><td>Original 26%</td><td>Modified 26%</td></tr>
<tr><td>In this study we are interested in whether a new app that uses your smartphone and smartwatch can encourage adults to increase their average level of activity on a weekly basis over a 12-month period. You have been asked to participate because you are an adult (40+ years old), are physically inactive, own a smart phone, and expressed interest in participating in this study. The research team hopes to recruit around 386 other participants in the study.</td><td>We want to know if a new digital health tool that uses your smartphone and smartwatch can help you to become more active. Our team is hoping to recruit 386 participants who are at least 25 years old, physically inactive,</td></tr>
</table>

		and own a smart phone. You were asked to participate because you expressed interest in the study.
**Preferences: Both versions emerged as good overall**		Feedback
**Both 38%**	Neither 10%	9

Benefits

Snippet #12: How will you be compensated?		
**Original 31%**	**Modified 33%**	
You will be rewarded points for reaching the daily step count goals, with 500 being the maximum number of points you can earn in a day. For every 500 points you earn, you will receive $1 from the research team, up to $20 per month or $240 during the 12-month period. The number of points assigned to each daily step goal will be based on the smartphone app, not by the research team, and on some days you will see a higher point value than other days. The amounts are calculated based on your answers to the evening survey and whether you have been reaching your step goal recently.	You will earn points for reaching your daily goals. Every 500 points earn you $1, up to $20/month and $240 for the 12 month study period. The reward amounts are controlled by the smartphone app and not by the research team.	
**Preferences: Both versions emerged as good overall**		Feedback
**Both 31%**	Neither 5%	11

Snippet #13: How will you be compensated?		
Original 21%	**Modified 40%**	
Twice during the study, you will also be asked to visit our lab to meet with a member of the research team. During the visits to our lab, you will be asked to complete a series of surveys, have a physical assessment, and complete a treadmill walking test --- the exact same tasks that you will have completed at the beginning of the study (taking up to 2 hours). You will receive a total of $90 for attending and completing each of the lab visits: $20 at the completion of the first visit, $30 at the completion of the 6-month visit, and $40 at the completion of the 12-month visit.	We will ask you to visit our lab a total of three times for 2 hour visits. You will be compensated after completing each of these visits: $20 for the first visit, $30 for the 6-month visit, and $40 for the 12-month visit (total $90). Every time, you will be asked to answer a few questions, have a physical assessment, and complete a walking test on a treadmill.	
**Preferences: Overall modified was preferred**		Feedback
Both 29%	Neither 10%	10

Snippet #15: How will you be compensated?	
**Original 52%**	Modified 5%

At the end of the study, you will be allowed to keep the Fitbit Versa if you attend all study visits and complete at least 80% of the daily survey questions through the app.	You can keep the Fitbit Versa at the end of the study, if you complete at least 80% of the daily surveys.	
**Preferences: Overall original was preferred**		Feedback
Both 40%	Neither 2%	9

Snippet #31: Will you receive any results from participating?		
**Original 43%**	Modified 26%	
You will not receive any individual results from any of the study measures, but a summary of the study results will be available at http://www.ClinicalTrials.gov. You may freely access this website at any time. There is no information that can be used to identify you on this website.	A summary of the study results will be available at http://www.ClinicalTrials.gov. We will not share any information that can personally identify you on this website.	
**Preferences: Overall original was preferred**		Feedback
Both 31%	Neither 0%	4

Procedure

Snippet #05: What will happen to you in this study?		
**Original 29%**	**Modified 37%**	
If the research team decides that you are eligible, you will be asked to do the following tasks at the UXXX Xxxxxxxx & Xxxxxxxx Xxxxxxxx Xxxxxxx Center (XXXXX). You should expect to spend about 2 hours at the XXXXX. You will complete several surveys about your physical activity, sleep, and other questions about your health and well-being. You will have a physical assessment, like visiting your doctor's office, which will include measuring your height, weight, and the circumference of your hips.	If you are eligible, you will be asked to spend about 2 hours at the UXXX Xxxxxxxx & Xxxxxxxx Xxxxxxxx Xxxxxxx Center (XXXXX). We will ask you questions about physical activity, sleep, and general well-being and will perform a physical exam, which will include measuring your height, weight, and hips.	
**Preferences: Both versions emerged as good overall**		Feedback
**Both 29%**	Neither 5%	12

Snippet #06: What will happen to you in this study?		
**Original 39%**	Modified 24%	
Medical professionals will collect a small droplet of blood from your finger to measure the amount of sugar in your blood. This may cause mild short-term pain and possibly bruising, and in rare cases fainting or a local infection.	We will measure your blood sugar level, which may cause mild pain, bruising, and in rare cases fainting or infection.	
**Preferences: Overall original was preferred**		Feedback
Both 21%	Neither 16%	16

Snippet #07: What will happen to you in this study?		
**Original 45%**	Modified 26%	
You will complete a treadmill walking test, while wearing a heart monitor on your chest, so that the research team can record your heart rate and blood pressure. The treadmill test will last approximately 5-10 minutes. The treadmill will start at a slow pace and gradually increase in speed and steepness, as though you are gradually walking faster up a hill. If you want to stop the test for any reason --- such as if you feel lightheaded, dizzy, or breathless --- a member of the research team will be there with you to help.	You will be asked to walk on a treadmill for 5-10 minutes, or as long as you can, while wearing a monitor on your chest to record your heart rate and blood pressure. A member of the research team will be there to help.	
**Preferences: Overall original was preferred**		Feedback
Both 24%	Neither 5%	13

Snippet #08: What will happen to you in this study?		
**Original 37%**	**Modified 8%**	
You will be given two physical activity monitors, including an ActiGraph and a Fitbit Versa. You will be required to download the Fitbit app to your smartphone.	We will give you an ACTi graph and Fitbit Versa to monitor your activity, and we will help you download a Fitbit app to your smartphone.	
**Preferences: Both versions emerged as good overall**		Feedback
**Both 45%**	Neither 11%	13

Snippet #09: What will happen to you in this study?	
**Original 29%**	**Modified 21%**
At this time you will be assigned at random to one of two participant groups, called the "control" group and the "intervention" group. Each group will receive slightly different daily activity (or step count) goals through the smartphone and Fitbit app during the 12-month study period. For example, if you walk 5,000 steps per day on average, then the highest goal you would expect from the smartphone app would be 10,000 steps per day. If you walk around 2,000 steps per day, then the highest goal you would receive would be 5,000 steps per day.	All participants will be split into two groups, called the "control" group and the "intervention" group. During the next 12 months, each group will receive slightly different daily goals through their smartphone and Fitbit app. The intervention group will receive personalized step goals, completing the daily survey on their watch, and completing a physical activity reflection and planning survey ~20 times over the year. The control group will only be getting a step goal of 10,000 steps/day and does not get the evening reflection survey or

		the physical activity planning survey.
**Preferences: Both versions emerged as good overall**		Feedback
**Both 37%**	Neither 13%	9

Snippet #10: What will happen to you in this study?	
**Original 42%**	Modified 21%
Every day during the first 7 days of the study, you will wear the ActiGraph as well as the Fitbit while you are awake. The ActiGraph will be worn with an elastic belt around your waist. At the end of the 7 days you will mail the ActiGraph back to the research team, using a paid postage envelope that we will provide you when you receive the activity monitors. If the ActiGraph is worn for less than 4 days, we may ask you to wear it for another week.	You will be asked to wear the ActiGraph and Fitbit for 7 days (during the day) at the beginning of the study. The ActiGraph is worn around the waist with an elastic belt. If you do not wear the ActiGraph for the full 7 days, we may ask you to keep it on for another week.

**Preferences: Overall original was preferred**		Feedback
Both 32%	Neither 5%	9

Snippet #11: What will happen to you in this study?	
**Original 34%**	**Modified 26%**
You will continue to wear the Fitbit every day during the 12-month period, while you are awake and when you are asleep. You will receive messages daily on your smartphone with a step count goal along with a very short survey on your Fitbit (1-3 questions) once in the morning and again at night. The daily step count goal will gradually increase during the 12-month period, to help you meet nationally recommended guidelines for daily activity.	For 12 months, you will be asked to wear the Fitbit every day, even while you are asleep (if you're willing). You will receive a new step count goal every day, which will gradually increase over time. You will also complete two very short surveys every day on your Fitbit (1-3 questions).

**Preferences: Both versions emerged as good overall**		Feedback
**Both 34%**	Neither 5%	10

Snippet #14: How much time will I spend on this study?	
**Original 50%**	Modified 14%
Over the 12-month study period, you will spend up to 12 hours participating in the study. This estimate includes the time spent during each of the three lab visits (at the beginning and then every 6 months) as well as about 10 minutes per-week completing study related questions through the smartphone app for 52 weeks.	12 hours in total, during the entire 12-month study period.

**Preferences: Overall original was preferred**		Feedback
Both 29%	Neither 7%	8

Snippet #23: What happens if you change your mind?		
**Original 24%**	**Modified 12%**	
You can choose to leave the study at any point. If you decide to stop participating, we ask you to notify our team and return your study devices. If we find any important information that may change how you feel about participating in the study, we will tell you about it.	At any point during the study, you can choose to no longer participate. If you decide to stop participating, we ask you to notify the study team and return your study device. If we find any important information that may change how you feel about participating in the study, we will tell you about it.	
**Preferences: Both versions emerged as good overall**		Feedback
**Both 60%**	Neither 5%	5

Snippet #28: Can you be removed from the study?		
**Original 31%**	**Modified 21%**	
Yes. You may be withdrawn from the study for the following reasons: (a) You develop a physical or mental health issue that prohibits compliance with the study's protocol, (b) you become pregnant, and (c) if you do not follow the instructions given you by the research team.	Yes. You may be withdrawn from the study for the following reasons: (a) You develop a physical or mental health issue, (b) you become pregnant, and (c) if you do not follow instructions given by the research team.	
**Preferences: Both versions emerged as good overall**		Feedback
**Both 40%**	Neither 7%	8

Snippet #30: Are there any costs associated with this study?		
**Original 43%**	Modified 24%	
There are no costs associated with this study. If your device is lost, stolen, or damaged you are responsible for contacting the study team who will address the issue on a case-by-case basis and replace it up to one time, if necessary.	No. If your device is lost, stolen, or damaged you are responsible for contacting the team.	
**Preferences: Overall original was preferred**		Feedback
Both 29%	Neither 5%	6

Snippet #32: Who can you call if you have questions?	
Original 21%	**Modified 43%**
If you find this explanation of this study insufficient and have additional questions, or any research-related problems, you may contact Dr. X at ###-###-####. If you would like to learn more about your rights as a research study participant or to report research-related problems, you may	If you have additional questions or research-related problems, you may contact Dr. X at ##-###-#### or the Human Research Protections

contact the Human Research Protections Program Office at ###-###-HRPP (###-###-####).		Program Office at ###-###-HRPP (###-###-####).
**Preferences: Overall modified was preferred**		Feedback
Both 24%	Neither 12%	4

Risks

Snippet #16: What are the possible risks?		
**Original 32%**		**Modified 24%**
Wearing the Fitbit may sometimes feel uncomfortable, but our staff will show you how to wear it, help you adjust if needed, and provide an (optional) tutorial.		Our staff will show you how to wear the Fitbit, so that it is comfortable. They will also give you a short tutorial about how to use the Fitbit and the Fitbit app, if you'd like.
**Preferences: Both versions emerged as good overall**		Feedback
**Both 37%**	Neither 8%	10

Snippet #17: What are the possible risks?		
**Original 34%**		**Modified 29%**
You may not want to answer all the survey questions. While we would like you to answer all the questions on each survey, we know you may feel anxious or embarrassed about answering some of them. It's OK to skip questions you do not want to answer or to stop participating at any time.		You may feel anxious or embarrassed about some of the survey questions. It's OK to skip questions or to stop participating at any time.
**Preferences: Both versions emerged as good overall**		Feedback
**Both 29%**	Neither 8%	11

Snippet #18: What are the possible risks?		
**Original 32%**		**Modified 37%**
As you become more active during the study, you may experience some discomfort, soreness, fatigue, etc., and particularly during the treadmill test. To minimize the risk, we will only ask you to walk. In addition, the smartphone app you will use is designed to increase your goals only gradually. Falling is also a risk during the treadmill test, so we will have someone next to the treadmill to help you if you lose your balance.		Becoming more active may lead to discomfort, soreness, and feeling tired. During the treadmill walking test there is a risk of falling, but we will have someone next to you to help you through the test.
**Preferences: Both versions emerged as good overall**		Feedback
**Both 21%**	Neither 11%	9

Snippet #19: What are the possible risks?	
**Original 26%**	**Modified 26%**

You may feel embarrassed or frustrated if you are unable to complete the treadmill test or to meet the daily step goals during the 12-month study period. Our team will try to minimize this risk by being positive and supportive.	Not being able to complete parts of the study, like daily step count goals or the treadmill test, may lead to feeling embarrassed or frustrated. We will try to minimize this risk by being positive and supportive.	
**Preferences: Both versions emerged as good overall**		Feedback
**Both 29%**	Neither 18%	7

Snippet #20: What are the possible risks?		
**Original 32%**	**Modified 26%**	
The most serious risk is that you may experience heart problems during the treadmill test. In order to minimize this risk, you will be monitored by trained experts with experience conducting over 1,000 similar exercise tests. The experts monitoring you during the treadmill test are also certified in CPR (Cardiopulmonary Resuscitation) and AED (Automated External Defibrillator), in case you do experience heart problems.	The most serious risk is that you may experience heart problems during the treadmill test. In order to minimize this risk, you will be monitored by experts who are certified in CPR (Cardiopulmonary Resuscitation) and AED (Automated External Defibrillator).	
**Preferences: Both versions emerged as good overall**		Feedback
**Both 37%**	Neither 5%	7

Snippet #21: What are the possible risks?		
**Original 45%**	Modified 13%	
If your risk of heart problems during the treadmill test is higher because of a pre-existing condition, we will ask your primary care physician to approve your participation in the study. During the treadmill test, we will fit you with an electrocardiogram to monitor your heartbeat and we will have a licensed doctor present to supervise, while you are on the treadmill. If an emergency call needs to be made, there is a telephone located in the lab.	If your risk of heart problems during the treadmill test is higher because of a pre-existing condition, we will ask you primary care physician to approve your participation in the study.	
**Preferences: Overall original was preferred**		Feedback
Both 34%	Neither 8%	8

Snippet #22: What are the possible risks?	
**Original 45%**	Modified 13%
There may also be some unknown risks that are currently unforeseeable. You will be informed of any significant new findings that may add risk. However, based on	You will be informed of any new information that may add risk.

existing information the benefits for participation outweigh the potential risks.		
**Preferences: Overall original was preferred**		Feedback
Both 29%	Neither 13%	8

Snippet #24: What if you are injured?		
**Original 26%**	**Modified 33%**	
If you are injured as a direct result of participating in this research, the University of XXXXXXXXXX will provide any medical care you need to treat those injuries. The University will not provide any other form of compensation to you if you are injured. You may call the Human Research Protections Program Office at ###-###-HRPP (###-###-####) for more information about this, to inquire about your rights as a research subject, or to report research-related problems.	If you are injured as a direct result of participating in this research, the University of XXXXXXXXXXX will provide any medical care you need, but not compensation. You may call the Human Research Protections Program Office at ###-###-HRPP (###-###-####) for more information.	
**Preferences: Both versions emerged as good overall**		Feedback
**Both 38%**	Neither 2%	7

Snippet #25: What are the possible risks?		
**Original 21%**	**Modified 29%**	
You will be assigned to a study group at random (by chance). Your assignment is based on chance rather than a medical decision made by the researchers. The study group you are assigned to might not be the group you would prefer to be in. Your assigned study group might also prove to be less effective or have more side effects than the other study groups(s), or other treatments available for your condition.	You will be randomly assigned to a study group, so there is a possible risk that you will not be assigned to the group you would prefer. There is also a risk that the group you are assigned proves to be less effective or have more side effects than the other study group.	
**Preferences: Both versions emerged as good overall**		Feedback
**Both 39%**	Neither 11%	9

Snippet #26: What if the Fitbit is lost, stolen, or damaged?		
**Original 33%**	**Modified 31%**	
Please let us know immediately if your Fitbit is lost, stolen or damaged and our team will respond on a case-by-case basis.	Please let us know immediately.	
**Preferences: Both versions emerged as good overall**		Feedback
**Both 26%**	Neither 10%	9

Snippet #27: How will your data and privacy be protected?	
**Original 45%**	Modified 24%

<table>
<tr><td colspan="2">We will take all legally allowed measures to keep your information confidential. These measures include: (a) Using an identification number instead of your name when we collect data for the study. Making sure the identification number is the only reference to your data, so your data cannot be linked to your name. (b) Storing all data on secure, password-protected servers and transferring data from Fitbit. (c) Ensuring that you are aware of Fitbit privacy policies and terms of service. Ensuring hat you can control and/or change your privacy settings. We will provide you with a handout prior to the start of your participation in the study on how to set your privacy preferences. (d) Keeping a master list of all participants and their informed consent in separate locked file cabinets. This way individuals are not easily connected to their electronic data or study results.</td><td colspan="2">How will your data and privacy be protected during the study? To keep your data confidential, we will use an identification number instead of your name, store all data on protected servers, and inform you of the Fitbit policies.</td></tr>
<tr><td colspan="3">Preferences: Overall original was preferred</td><td>Feedback</td></tr>
<tr><td>Both 24%</td><td colspan="2">Neither 7%</td><td>10</td></tr>
</table>
